# Survival Outcomes in Metastatic Ewing Sarcoma Treated With Whole-Lung Radiation

**DOI:** 10.7759/cureus.26750

**Published:** 2022-07-11

**Authors:** Alexa Dang, Xiaolan Feng, Jeremy Hamm, Caroline L Holloway, Pauline T Truong

**Affiliations:** 1 Radiation Oncology, BC Cancer - Vancouver Centre, Vancouver, CAN; 2 Medical Oncology, BC Cancer - Vancouver Island Centre, Victoria, CAN; 3 Cancer Surveillance and Outcomes - Data and Analytics, BC Cancer - Vancouver Centre, Vancouver, CAN; 4 Radiation Oncology, BC Cancer - Vancouver Island Centre, Victoria, CAN

**Keywords:** overall survival, pulmonary metastases, metastatic, whole lung radiation, ewing sarcoma

## Abstract

Background and objective

There is a scarcity of research on outcomes in patients with metastatic Ewing sarcoma limited to pulmonary metastases who receive whole-lung radiotherapy (WLRT). In light of this, this study aimed to evaluate the use of WLRT and compare the survival outcomes between patients with metastatic Ewing sarcoma who received treatment with WLRT and those who did not.

Materials and methods

Patients of all ages with metastatic Ewing sarcoma restricted to the lung who were referred to the British Columbia (BC) Cancer from 1995 to 2017 were identified from the Sarcoma Outcomes Unit (SARCOU). Patient demographics and tumor and treatment characteristics were compared between cohorts treated with WLRT versus those who did not undergo WLRT. Five-year progression-free survival (PFS) and overall survival (OS) were evaluated using Kaplan-Meier (KM) estimates and compared between treatment groups with log-rank tests.

Results

The study cohort comprised 30 patients (median follow-up time: 6.8 years). Overall, the median age of the patients was 16 years (range: 4-86 years) and 60% were female; the primary disease sites were as follows: 27% axial skeleton, 53% appendicular skeleton, 20% visceral, 86% had ≥2 lung metastases, and 60% had bilateral disease. Fifteen (50%) patients received WLRT (median of 1500 cGy in 10 fractions). Chemotherapy was used in 97% of patients. The rate of surgery for lung metastases was 40%, which was similar between the WLRT and non-WLRT groups. The median size of the largest lung metastasis in the WLRT cohort was 1 cm (range: 0.3-1.8 cm), compared to 2 cm (range 0.5-6.7 cm) in the non-WLRT cohort (p=0.05). Demographics and tumor characteristics were otherwise not significantly different between the two treatment groups (all p>0.05). Among patients who received WLRT, 53% had complete response (CR), 7% partial response (PR), and 40% had disease progression. The five-year PFS was 86% vs. 59% (p=0.33) and OS was 78% vs. 54% (p=0.24) respectively for patients in the WLRT group vs. those in the non-WLRT group. The five-year PFS outcomes were higher on univariate analysis in patients with appendicular skeletal compared to axial skeletal and visceral primary sites (87.5% vs. 58% vs. 50%, respectively, p=0.02) and in patients with the size of the largest lung metastasis <2 cm vs. those with a size ≥2 cm (80% vs. 25%, p=0.04).

Conclusions

Patients treated with WLRT had a smaller-volume lung disease and over half of the patients who received WLRT had either complete or partial response. Trends of improved PFS and OS at five years were observed among patients who received WLRT compared to the non-WLRT group, but these were not statistically significant.

## Introduction

Ewing sarcoma is the second most common bone malignancy in the pediatric age group and adolescents but it can occur at any age. The landscape of treatment options has evolved significantly over the past decades with the intensification of systemic treatment regimens, in combination with local treatments with surgery and radiotherapy (RT), reflecting the importance of a multidisciplinary approach for these patients [1]. Despite the advances in treatment, outcomes in patients with metastatic disease remain poor. Ewing tumors can present as metastatic disease in up to 25% of cases with only 10% of patients presenting with metastasis restricted to the lung at diagnosis [1-3]. There is limited research on outcomes in patients with metastatic Ewing sarcoma limited to lung metastases after receiving whole-lung radiotherapy (WLRT). Previous studies have reported that patients with metastases restricted to the lung have better event-free and overall survival (OS) compared to patients with metastatic disease in other sites [1,4,5]. Clinical outcomes and overall toxicity secondary to whole-lung radiation remain unclear despite this modality being included in multiple guidelines as the standard treatment worldwide [6,7]. This study aims to evaluate the rate of use of WLRT and local control and survival outcomes in patients with Ewing sarcoma with metastasis limited to the lung treated at the British Columbia (BC) Cancer.

## Materials and methods

Inclusion and exclusion criteria

Patients of all ages with metastatic Ewing sarcoma restricted to the lung who were treated at BC Cancer from 1995 to 2017 were identified using the BC Cancer Registry and the Sarcoma Outcomes Unit (SARCOU). Patients with de novo lung metastasis as well as those who developed lung metastasis after primary Ewing sarcoma diagnosis were included. The subset of patients who received whole-lung radiation for lung metastases was the focus of this study. Patients who received palliative lung radiation or those who had extrapulmonary metastases prior to the development of pulmonary metastases were excluded.

Study design and ethical approval

This study employed a single cohort retrospective design. The study was approved by the University of British Columbia/BC Cancer Research Ethics Board (study number: H19-02506).

Data collection

A chart review was performed to abstract data on patient demographics and tumor and treatment characteristics. Treatment response was retrospectively assessed based on available documentation and imaging by using the response evaluation criteria in solid tumors (RECIST) version 1.1.

Statistical analysis

Descriptive analysis was used to analyze patient, tumor, and treatment factors in the entire cohort, which were compared between subgroups treated with WLRT and those who did not receive WLRT by using chi-square tests. The primary outcome was OS and the secondary outcome was progression-free survival (PFS). OS was calculated from the date of diagnosis till death from any cause. Survival outcomes were analyzed using the Kaplan-Meier (KM) method and compared between subgroups using log-rank tests. Patients were censored at the time of the last follow-up visit. Statistical significance was established at p<0.05. All statistical analysis was performed using Statistical Analysis Software (SAS) Version 9.4 for Microsoft Windows (SAS Institute, Cary, NC).

## Results

Demographics

Of the total 182 patients with Ewing sarcoma identified by SARCOU, 30 had pulmonary metastasis only. Within the cohort of 30 patients, 15 (50%) received WLRT. Eighteen patients in the cohort were aged <18 years, 10 of whom were in the WLRT group. Overall, the median age was 16 years (range: 4-86 years) and 60% of patients were female. The primary disease sites were as follows: 27% axial skeleton, 53% appendicular skeleton, and 20% visceral, with 60% having bilateral lung disease and 86% having two or more lung metastases (Table 1). Regarding treatments received, 77% of patients had surgery to the primary and 23% did not. Within the surgical series, 96% received induction chemotherapy, 48% received neoadjuvant RT, 35% received adjuvant chemotherapy, and 26% received adjuvant RT. As for the patients who did not receive surgery for their primary disease, 71% received induction chemotherapy, 14% received neoadjuvant RT, and 71% received definitive RT. Regarding the treatment of lung metastases specifically, 97% underwent standard chemotherapy and 40% had surgery for the lung metastasis. The median size of the largest lung metastasis in the WLRT cohort was 1 cm (range: 0.3-1.8 cm) compared to 2 cm (range 0.5-6.7 cm) in the non-WLRT cohort (p=0.05). Demographics and tumor characteristics were otherwise similar between the two treatment groups (all p>0.05).

**Table 1:** Clinical and treatment characteristics in the overall cohort and comparisons between WLRT and non-WLRT cohorts WLRT: whole-lung radiotherapy; RT: radiotherapy

Variables		Overall	WLRT	Non-WLRT	P-value
		N=30	N=15	N=15	
		Age at diagnosis (years)	Median (range)	16 (4-86)		15 (5-53)	17 (4-86)	0.27
			<30, n (%)	24 (80%)		13 (87%)	11 (73%)	0.65
	≥30, n (%)	6 (20%)	2 (13%)	4 (27%)	
Gender					
	Male, n (%)	12 (40%)	6 (40%)	6 (40%)	1
	Female, n (%)	18 (60%)	9 (60%)	9 (60%)	
Primary disease site					
	Axial skeleton, n (%)	8 (27%)	4 (27%)	4 (27%)	0.17
	Appendicular skeleton, n (%)	16 (53%)	10 (67%)	6 (40%)	
	Visceral, n (%)	6 (20%)	1 (7%)	5 (33%)	
Diagnosis of lung metastasis					
	Pathological, n (%)	13 (46%)	7 (47%)	6 (46%)	0.55
	Clinical, n (%)	15 (54%)	8 (53%)	7 (54%)	
Laterality of lung metastasis					
	Unilateral, n (%)	10 (40%)	3 (25%)	7 (54%)	0.31
	Bilateral, n (%)	15 (60%)	9 (75%)	6 (46%)	
Number of lung metastasis	Solitary, n (%)	4 (14%)	1 (7%)	3 (21%)	0.84
	2-5, n (%)	10 (36%)	5 (36%)	5 (36%)	
	>5, n (%)	14 (50%)	8 (57%)	6 (43%)	
Size of largest lung metastasis (cm)	Median (range)	1 (0.3-6.7)	1 (0.3-1.8)	2 (0.5-6.7)	0.05
Neoadjuvant RT to primary	N (%)	12 (40%)	7 (47%)	5 (33%)	0.71
Induction chemotherapy	N (%)	27 (90%)	15 (100%)	12 (80%)	0.22
Surgery to primary	N (%)	23 (77%)	14 (93%)	9 (60%)	0.08
Definitive RT to primary	N (%)	5 (17%)	1 (7%)	4 (27%)	0.33
Adjuvant RT to primary	N (%)	6 (20%)	5 (33%)	1 (7%)	0.17
Adjuvant chemotherapy	N (%)	9 (28%)	7 (47%)	2 (13%)	0.11
Chemotherapy for lung metastasis	N (%)	29 (97%)	15 (100%)	14 (93%)	1
Surgery for lung metastasis	N (%)	12 (40%)	6 (40%)	6 (40%)	1

Treatment

The mean dose of WLRT was 1500 cGy in 10 fractions (range: 1200 cGy in eight fractions to 1800 cGy in 12 fractions). Among patients who received WLRT, 53% had complete response (CR), 7% partial response (PR), and 40% had disease progression (Table 2). In patients with de novo lung metastasis at diagnosis, 50% vs. 33% had CR, 30% vs. 0% had PR, and 20% vs. 67% had disease progression on chemotherapy in the WLRT vs. non-WLRT groups respectively (p=0.2). In patients who developed lung metastasis after diagnosis, 20% vs. 25% had CR, none had PR, 40% vs. 0% had no response (NR), and 40% vs. 63% had progression with first-line chemotherapy in the WLRT vs. non-WLRT groups (p=0.3). Patients treated with de novo lung metastasis received standard chemotherapy per Children’s Oncology Group (COG) guidelines or institutional protocols consisting of cyclophosphamide, vincristine, doxorubicin, ifosfamide-mesna, and etoposide [8,9].

**Table 2:** Response of lung metastasis to chemotherapy and WLRT *Chemotherapy regimen of alternating etoposide, ifosfamide-mesna (SAIME) with vincristine, doxorubicin, and cyclophosphamide (SAVAC) WLRT: whole-lung radiotherapy; CR: complete response, PR: partial response, NR: no response, NA: not applicable

	Overall, n (%)	WLRT, n (%)	Non-WLRT, n (%)	P-value
Response of de novo lung metastasis to first-line chemotherapy*				
CR	7 (44%)	5 (50%)	2 (33%)	0.17
PR	3 (19%)	3 (30%)	0 (0%)	
NR	0 (0%)	0 (0%)	0 (0%)	
Progression	6 (37%)	2 (20%)	4 (67%)	
Response of lung metastasis progression to first-line chemotherapy*				
CR	3 (23%)	1 (20%)	2 (25%)	0.29
PR	0 (0%)	0 (0%)	0 (0%)	
NR	2 (15%)	2 (40%)	0 (0%)	
Progression	7 (54%)	2 (40%)	5 (63%)	
Response of lung metastasis to WLRT				
CR	NA	8 (53%)	NA	NA
PR	NA	1 (7%)	NA	
NR	NA	0 (0%)	NA	
Progression	NA	6 (40%)	NA	

Outcomes

The five-year PFS was 86% vs. 59% (p=0.33) and OS was 78% vs. 54% (p=0.24) respectively for patients treated with WLRT vs. those who did not receive WLRT (Table 3). The five-year PFS outcomes were higher on univariate analysis in patients with appendicular skeletal compared to axial skeletal and visceral primary sites (87.5% vs. 58% vs. 50%, respectively, p=0.02) and in patients with the size of largest lung metastasis <2 cm vs. those with a size ≥2 cm (80% vs. 25%, p=0.04).

**Table 3:** Five-year Kaplan-Meier (KM) PFS and OS according to clinical and treatment characteristics PFS: progression-free survival; OS: overall survival; WLRT: whole-lung radiotherapy; NA: not applicable

		5-year KM PFS	Log rank p	5-year KM OS	Log rank p
						Overall		0.73		0.67	
Age at diagnosis (years)	<30	0.74	0.17	0.68	0.19
	≥30	0.67		0.6	
Gender	Male	0.65	0.95	0.73	0.63
	Female	0.78		0.63	
Primary disease site	Axial skeleton	0.58	0.02	0.71	0.14
	Appendicular skeleton	0.88		0.74	
	Visceral	0.5		0.4	
Laterality of lung metastasis	Unilateral	0.69	0.51	0.63	0.95
	Bilateral	0.73		0.66	
Number of lung metastasis	<5	0.8	0.18	0.78	0.34
	≥5	0.64		0.56	
Size of largest lung metastasis (cm)	<2	0.8	0.04	0.71	0.44
	≥2	0.25		0.33	

The overall median survival and PFS were 6.8 years and 6.2 years respectively (Table 4).

**Table 4:** Median survival and PFS in patients treated with WLRT vs. those without WLRT: whole-lung radiotherapy; PFS: progression-free survival

		Overall (n=30)	WLRT	Non-WLRT
					Survival (years)	Median	6.8	6.8	4.8
					PFS (years)	Median	6.2	6.2	6.6

Toxicity

One patient developed a chest wall myofibrosarcoma thought to be secondary to radiation to the chest wall at the site of primary disease as opposed to WLRT. Pulmonary function tests (PFTs) were not routinely used in follow-ups after WLRT. No cases of pneumonitis were reported. Within the WLRT group, two patients had reported chronic shortness of breath thought to be secondary to anthracycline use but had reduced respiratory capacity and obstructive airway changes. The second patient had a history of pre-existing reactive airway disease prior to the treatment requiring puffers. Worsening chronic shortness of breath was thought to be a combination of WLRT and bleomycin; however, PFTs were relatively stable pre- and post-treatment.

## Discussion

Ewing sarcoma is a radiosensitive tumor; while chemotherapy and radiation play an integral role in its treatment, the impact of WLRT on lung disease on patient outcomes is less well defined [10-12]. WLRT has been employed since the 1970s in the metastatic setting [13]. The German Cooperative Ewing Sarcoma Studies (CESS) looked at survival in patients with pulmonary metastasis at diagnosis who were treated with WLRT [14]. Of the 30 evaluable patients, 29 had complete radiographic remission following chemotherapy or resection of lung metastases. Within the same group, 22 patients underwent WLRT. Ten patients achieved complete remission, of which nine had received WLRT. Intergroup Ewing’s Sarcoma Studies of Metastatic Disease showed 30% remission at three years in patients receiving WLRT [15].

Our results of trends toward increased PFS and OS with WLRT are supported by the literature. The European Intergroup Cooperative Ewing’s Sarcoma Studies (EICESS) showed a statistically significant increased four-year event-free survival (EFS) of 40% vs. 19% with WLRT with total doses between 15-18 Gy; however, patient selection criteria for WLRT were not clear [16]. Studies have employed response to chemotherapy as a means of offering WLRT including WLRT after incomplete pulmonary response after initial chemotherapy and, alternatively, WLRT if complete remission is achieved following primary chemotherapy [17,18]. A survival analysis from the EICESS group looked at 114 patients with metastatic Ewing sarcoma with pulmonary and/or pleural metastases, 75 of whom received WLRT [17]. The reported five-year EFS was 38% with WLRT vs. 27% without WLRT after neoadjuvant systemic and local therapy to the primary tumor (p=0.002). The rate of pulmonary relapse was 20% with WLRT vs. 40% without WLRT (p=0.046). Risk factors on multivariate analysis associated with poor prognosis included the poor response of the primary tumor to chemotherapy and bilateral lung metastases [17]. Bölling et al. documented five-year OS of 61% with WLRT and 49% without WLRT, although not statistically significant along with similar rates of five-year EFS of 39% with WLRT and 37% without WLRT [19]. Pulmonary relapse rates between groups were not significantly different (48.5% with WLRT vs. 38.8% without WLRT, p=0.46) and are comparable with our results.

A number of studies have identified negative prognostic factors including poor radiographic response to the primary tumor, and incomplete radiographic response of lung disease after induction treatment to the primary and bilateral metastatic pulmonary involvement [17,20]. Casey et al. showed three-year EFS, OS, and freedom from pulmonary relapse to be 38%, 45%, and 45% respectively for adult patients receiving WLRT [4]. Our cohort had higher survival rates than reported in the literature for lung-only metastasis, possibly due to smaller lung volume owing to disease selection bias. Based on the benefits seen in the above studies, WLRT has become part of the standard of care for patients with metastatic Ewing sarcoma to the lungs after completing chemotherapy and local treatment to the primary disease.

WLRT is associated with risks including mild hypoplasia of the chest wall and thoracic spine, pneumonitis, pulmonary fibrosis, reduced lung capacity, hypothyroidism, and the rare but serious risk of secondary malignancy in the treated field including thyroid, lung, bone, breast, and soft tissue [11,19]. The effects of prophylactic lung irradiation in osteosarcoma on spirometry have been shown to lead to a minimal decrease in forced vital capacity and forced expiratory volume up to one year post-WLRT that normalize by two years post-treatment [21]. In the current study, no significant toxicities were observed following WLRT with no cases of pneumonitis or secondary malignancies reported. Two patients experienced chronic shortness of breath following treatment, attributed to the combination with chemotherapy including anthracycline and bleomycin. The risk of cardiac toxicity can also be influenced by both thoracic radiation and chemotherapy, which may increase the risk of treatment-associated cardiomyopathy [15]. A systematic review by Ronchi et al. showed reported rates of ≥3-grade acute lung toxicity associated with WLRT ranging from 0 to 12%, specifically a 1.8% risk of severe pneumonitis [6]. Many studies have shown that WLRT is well tolerated with limited toxicity and our results are consistent with these [4,10,13,19]. Toxicity is dependent on the total lung dose, which should not exceed 18-20 Gy [13]. Although the toxicities of WLRT are well studied in the pediatric population, there is limited data among adult patients [22]. In BC, pediatric as well as adolescent and young adult survivors are followed into adulthood to monitor for long-term adverse events related to WLRT. Given the retrospective nature of the study and the large part of the cohort being pediatric and treated at a separate health authority, a timed measurement of lung function for follow-up was not routinely performed.

Our study is limited by its retrospective nature and small sample size. With retrospective studies, thorough descriptions of toxicity in the long term are difficult to capture. No clear selection criteria for WLRT candidates were reported; however, previous significant thoracic primary doses did preclude patients from receiving WLRT. Although this was a retrospective study, all cases were discussed at a multidisciplinary conference as part of a provincial standard and this study contributes real-world data from a population-based cohort. The future areas of study include the role of stereotactic RT in oligometastatic or oligoprogressive disease, metastatectomy as well as targeted therapies in the setting of oncogene proteins and tumor environment [23]. Interpretation of the contribution of specific therapies to outcomes can be challenging given that these patients often receive multi-modality care, which emphasizes the importance of a multi-disciplinary approach for these patients.

## Conclusions

Our results are consistent with findings in the available literature suggesting a benefit of WLRT in select patients. Trends for improved PFS and OS at five years were observed among patients who received WLRT, but these were not statistically significant. Patients treated with WLRT had smaller-volume lung disease and the majority had a complete or partial response with low rates of toxicity reported.

## References

[1] Rodríguez-Galindo C, Liu T, Krasin MJ (2007). Analysis of prognostic factors in Ewing sarcoma family of tumors: review of St. Jude Children's Research Hospital studies. Cancer.

[2] Paulussen M, Bielack S, Jürgens H, Jost L (2008). Ewing's sarcoma of the bone: ESMO clinical recommendations for diagnosis, treatment and follow-up. Ann Oncol.

[3] Huang M, Lucas K (2011). Current therapeutic approaches in metastatic and recurrent Ewing sarcoma. Sarcoma.

[4] Casey DL, Alektiar KM, Gerber NK, Wolden SL (2014). Whole lung irradiation for adults with pulmonary metastases from Ewing sarcoma. Int J Radiat Oncol Biol Phys.

[5] Cotterill SJ, Ahrens S, Paulussen M, Jürgens HF, Voûte PA, Gadner H, Craft AW (2000). Prognostic factors in Ewing's tumor of bone: analysis of 975 patients from the European Intergroup Cooperative Ewing's Sarcoma Study Group. J Clin Oncol.

[6] Ronchi L, Buwenge M, Cortesi A (2018). Whole lung irradiation in patients with osteosarcoma and Ewing sarcoma. Anticancer Res.

[7] (2021). National Cancer Institute: Ewing sarcoma treatment (PDQ). http://www.cancer.gov/cancertopics/pdq/treatment/ewings/HealthProfessional/page6.

[8] Womer RB, West DC, Krailo MD (2012). Randomized controlled trial of interval-compressed chemotherapy for the treatment of localized Ewing sarcoma: a report from the Children's Oncology Group. J Clin Oncol.

[9] Jain S, Kapoor G (2010). Chemotherapy in Ewing's sarcoma. Indian J Orthop.

[10] Raciborska A, Bilska K, Rychłowska-Pruszyńska M (2016). Management and follow-up of Ewing sarcoma patients with isolated lung metastases. J Pediatr Surg.

[11] Yang JC, Wexler LH, Meyers PA, Happersett L, La Quaglia MP, Wolden SL (2013). Intensity-modulated radiation therapy with dose-painting for pediatric sarcomas with pulmonary metastases. Pediatr Blood Cancer.

[12] Pinkerton CR, Bataillard A, Guillo S, Oberlin O, Fervers B, Philip T (2001). Treatment strategies for metastatic Ewing's sarcoma. Eur J Cancer.

[13] Whelan JS, Burcombe RJ, Janinis J, Baldelli AM, Cassoni AM (2002). A systematic review of the role of pulmonary irradiation in the management of primary bone tumours. Ann Oncol.

[14] Dunst J, Paulussen M, Jürgens H (1993). Lung irradiation for Ewing's sarcoma with pulmonary metastases at diagnosis: results of the CESS-studies. Strahlenther Onkol.

[15] Cangir A, Vietti TJ, Gehan EA (1990). Ewing's sarcoma metastatic at diagnosis. Results and comparisons of two intergroup Ewing's sarcoma studies. Cancer.

[16] Paulussen M, Ahrens S, Burdach S (1998). Primary metastatic (stage IV) Ewing tumor: survival analysis of 171 patients from the EICESS studies. European Intergroup Cooperative Ewing Sarcoma Studies. Ann Oncol.

[17] Paulussen M, Ahrens S, Craft AW (1998). Ewing's tumors with primary lung metastases: survival analysis of 114 (European Intergroup) Cooperative Ewing's Sarcoma Studies patients. J Clin Oncol.

[18] Spunt SL, McCarville MB, Kun LE, Poquette CA, Cain AM, Brandao L, Pappo AS (2001). Selective use of whole-lung irradiation for patients with Ewing sarcoma family tumors and pulmonary metastases at the time of diagnosis. J Pediatr Hematol Oncol.

[19] Bölling T, Schuck A, Paulussen M (2008). Whole lung irradiation in patients with exclusively pulmonary metastases of Ewing tumors. Toxicity analysis and treatment results of the EICESS-92 trial. Strahlenther Onkol.

[20] Luksch R, Tienghi A, Hall KS (2012). Primary metastatic Ewing's family tumors: results of the Italian Sarcoma Group and Scandinavian Sarcoma Group ISG/SSG IV Study including myeloablative chemotherapy and total-lung irradiation. Ann Oncol.

[21] Ellis ER, Marcus RB Jr, Cicale MJ (1992). Pulmonary function tests after whole-lung irradiation and doxorubicin in patients with osteogenic sarcoma. J Clin Oncol.

[22] Tanguturi SK, George S, Marcus KJ, Demetri GD, Baldini EH (2015). Whole lung irradiation in adults with metastatic Ewing sarcoma: practice patterns and implications for treatment. Sarcoma.

[23] Gaspar N, Hawkins DS, Dirksen U (2015). Ewing sarcoma: current management and future approaches through collaboration. J Clin Oncol.

